# The Importance of the Mixing Energy in Ionized Superabsorbent Polymer Swelling Models

**DOI:** 10.3390/polym12030609

**Published:** 2020-03-07

**Authors:** Eanna Fennell, Juliane Kamphus, Jacques M. Huyghe

**Affiliations:** 1 Bernal Institute, University of Limerick, Castletroy, V94 T9PX Limerick, Ireland; eanna.fennell@ul.ie; 2 School of Engineering, University of Limerick, Castletroy, V94 T9PX Limerick, Ireland; 3 Procter & Gamble Service GmbH, Sulzbacher Straße 40, 65824 Schwalbach am Taunus, Germany; kamphus.j@pg.com; 4 Department of Mechanical Engineering, Eindhoven University of Technology, 5600 MB Eindhoven, The Netherlands

**Keywords:** ionized hydrogels, swelling, mixing energy, Flory–Rehner theory, finite deformation, Flory–Huggins equation, sodium polyacrylate, superabsorbent polymer, polymer mechanics

## Abstract

The Flory–Rehner theoretical description of the free energy in a hydrogel swelling model can be broken into two swelling components: the mixing energy and the ionic energy. Conventionally for ionized gels, the ionic energy is characterized as the main contributor to swelling and, therefore, the mixing energy is assumed negligible. However, this assumption is made at the equilibrium state and ignores the dynamics of gel swelling. Here, the influence of the mixing energy on swelling ionized gels is quantified through numerical simulations on sodium polyacrylate using a Mixed Hybrid Finite Element Method. For univalent and divalent solutions, at initial porosities greater than 0.90, the contribution of the mixing energy is negligible. However, at initial porosities less than 0.90, the total swelling pressure is significantly influenced by the mixing energy. Therefore, both ionic and mixing energies are required for the modeling of sodium polyacrylate ionized gel swelling. The numerical model results are in good agreement with the analytical solution as well as experimental swelling tests.

## 1. Introduction

An ionized hydrogel is a fluid-filled amorphous cross-linked network of polymer chains with an associated ionic charge. The ability of these structures to deform up to orders of magnitude greater than their original volume through the absorption of fluid gives rise to a variety of applications [1]. These applications include the design of biosensors, moisture retention in soil and cement, wound dressings and industrial hygiene products (e.g., sanitary towels and diapers) [2,3,4]. The environmental conditions, such as the temperature, pH and ionic strength, of each of these applications plays a critical role in the volume of fluid absorbed by the gel [5,6]. As a result of the myriad of variables involved in ionized hydrogel swelling, representative numerical modeling is essential to replicate the swelling process for optimal efficacy in product design.

There have been many published studies modeling the swelling of gels [7]. Each of these models revolve around the Flory–Rehner (FR) theory of an ideal elastomeric gel which states the total free energy in the system is a sum of the elastic, mixing and ionic energies [8,9]. Successful recent work on reaching large deformation with the finite element method have excelled with the addition of the Terzaghi decomposition to split the stress in the system into the effective stress acting on the solid matrix and the pressure of the fluid in the pores [10,11]. Three-field finite element formulations which solve for position, chemical potential and fluid flux independently, have shown greater stability than two-field formulations [12,13,14]. The assumption of perfect separability of the energies of FR theory has been questioned in several studies. Mostly, the presence of the charged groups on the hydrophilic polymer chains in ionized gels effects the polymer/solvent interaction and hence the mixing energy (ionic/mixing dependency) [15]. Several studies have shown the interdependency of the elastic and mixing energies (elastic/mixing dependency) [16,17]. Finally, the ionic concentration of the external solution was shown to influence the modulus of a confined pHEMA gel at constant strain (elastic/ionic dependency) [18]. However, numerical simulation has shown the relatively small effect that assuming perfect separability has on gel swelling and as an alternative theory is yet to be proposed, the following study assumes Flory–Rehner to be sufficient in describing hydrogel swelling [19].

**Figure 1:**
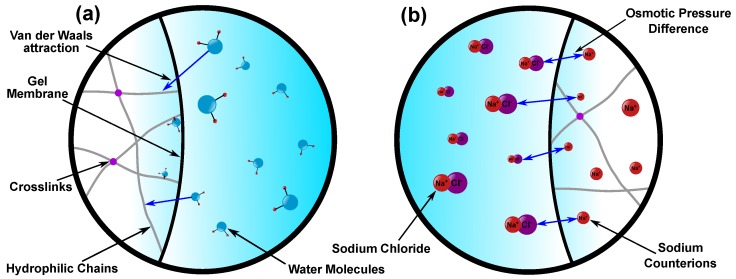
(**a**) Mixing Energy: attraction of the solvent (water) molecules in the external solution towards the hydrophilic polymer chains causing fluid flow into the gel. (**b**) Ionic Energy: osmotic pressure gradient resulting from the molar concentration difference between the sodium counterions inside the gel and the ionic composition of the external solution. This drives fluid either into or out of the gel depending on the sign of the osmotic pressure gradient.

Within this theoretical framework, the mixing and ionic energies contribute to swelling, whereas the elastic energy ensures large deformation of the polymer chains is not possible. The mixing energy describes the attraction of solvent molecules towards the hydrophilic polymer chains through a weak Van der Waals force (Figure 1a). The ionic energy can be defined as the difference in ionic concentration across the semi-permeable membrane of the gel which results in an osmotic pressure (Figure 1b). For non-ionized gels (e.g., polyvinyl alcohol, polyacrylamide, etc.), the ionic contribution to swelling can be neglected and be fully modeled using just the mixing energy [11]. On the other hand, there is more uncertainty surrounding ionized gels. Experiments on swelling sodium polyacrylate gels showed that the mixing energy can be assumed negligible when swelling equilibrium is reached [20,21,22,23]. Several studies have used this assumption to develop swelling models of ionized gels [10,12,24,25] as well as biological tissue [26,27,28]. However, the effect of the mixing energy during transient swelling is not documented experimentally due to the difficulty of dynamically splitting the swelling pressures.

Therefore, the magnitude of the mixing energies contribution to the swelling of ionized hydrogels has not been clearly defined. In this study, the effect of each energy on hydrogel swelling in univalent and divalent solutions is investigated. Furthermore, the effect of including the mixing energy on transient surface instability magnitude is examined. A mixed hybrid finite element method is utilized to model the phenomenon under physiological solution concentrations at varying initial solid volume fractions. The model results are exhaustively validated by comparing back to the theoretical predictions of the swelling pressure and finally to experimental swelling tests.

## 2. Methods

### 2.1. Swelling Model

#### 2.1.1. Equilibrium Conditions

Thermodynamic equilibrium is reached when the difference between the chemical potential inside and outside the gel ($\mu_{i}$) is zero,

(1) $$\begin{array}{c} {\bar{\mu }}_{f,gel}-{\bar{\mu }}_{f,sol}=0, \end{array}$$

where f denotes the solvent in the gel and the solution (sol). Equation (1) can be expressed in terms of the total pressure ($\Pi_{total}$) in the system,

(2) $$\begin{array}{c} \Pi_{total}=-\frac{{\bar{\mu }}_{f,gel}-{\bar{\mu }}_{f,sol}}{{\bar{V}}_{f}}=0, \end{array}$$

where ${\bar{V}}_{f}$ is the molar volume of the solvent. The total pressure in a swelling hydrogel can be divided into three separate parts using the Flory–Rehner (FR) theory of an ideal elastomeric gel. This theory has been implemented in a multitude of hydrogel swelling models and states the perfect separability of the total free energy, (∆F${}_{total}$), into an elastic (∆F${}_{elastic}$), mixing (∆F${}_{mixing}$) and ionic (∆F${}_{ionic}$) contribution, each with an associated pressure ($\Pi_{elastic}$, $\Pi_{mixing}$ and $\Pi_{ionic}$),

(3) $$\begin{array}{c} ∆F_{total}=∆F_{ionic}+∆F_{mixing}+∆F_{elastic}. \end{array}$$

when Equation (1) equals zero, the gel is considered to be in equilibrium. The mixing and ionic contributions are commonly seen as the cause of gel swelling whereas the elastic portion restricts the large expansion of the material. Therefore, the total swelling pressure ($\Pi_{swelling}$) can be defined as,

(4) $$\begin{array}{c} \Pi_{swelling}=\Pi_{ionic}+\Pi_{mixing}. \end{array}$$

#### 2.1.2. Mixing Energy

The mixing energy refers to the attraction of solvent molecules in the external solution to the hydrophilic polymer chains and can be described by the Flory–Huggins equation [29,30],

(5) $$\begin{array}{c} \Pi_{mixing}=-\frac{RT}{{\bar{V}}_{f}}\left(ln\left(1-\phi_{s}\right)+\phi_{s}+\chi_{0}\phi_{s}^{2}+\chi_{1}\phi_{s}^{3}\right), \end{array}$$

where *R* is the universal gas constant, *T* is the temperature, $\phi_{s}$ is the current solid volume fraction and χ${}_{0}$ and χ${}_{1}$ are Flory–Huggins parameters derived from the solid-fluid interaction parameter, $\chi =\chi_{0}+\chi_{1}\phi_{s}$. These parameters are material and environment dependent and de-swelling experiments assuming FR theory are implemented to calculate the Flory–Huggins parameters [20].

#### 2.1.3. Ionic Energy

The ionic energy is associated with the osmotic pressure difference between the inside and the outside the gel as a result of a molar concentration gradient. Differentiating this energy (∆F${}_{ion}$) with respect to the volume fractions of the fluid ($\phi_{f}$), positive ions and negative ions results in the total ionic osmotic pressure,

(6) $$\begin{array}{c} ∆F_{ionic}\left(\phi_{f},\phi_{+},\phi_{-}\right)=\mu_{f,0}\phi_{f}+\mu_{+,0}\phi_{+}+\mu_{-,0}\phi_{-}-RT\Gamma \left(\frac{\phi_{+}}{{\bar{V}}_{+}}+\frac{\phi_{-}}{{\bar{V}}_{-}}\right)ln\left(\phi_{f}\right)\\ +RT\frac{\phi_{+}}{{\bar{V}}_{+}}\left(ln\frac{\phi_{+}}{{\bar{V}}_{+}}-1\right)+RT\frac{\phi_{-}}{{\bar{V}}_{-}}\left(ln\frac{\phi_{-}}{{\bar{V}}_{-}}-1\right), \end{array}$$

where Γ are the osmotic coefficients and c is the concentration of the fluid, positive ions and negative ions respectively (f, +, -). However, as the ion phases are largely fast compared to the flux of the solvent into the gel, the contribution of the ions are ignored [10]. Therefore, the ionic osmotic pressure is calculated from,

(7) $$\begin{array}{c} \Pi_{ionic}=-\frac{\partial ∆F_{ionic}}{\partial \phi_{f}}. \end{array}$$

This yields an osmotic pressure difference between inside and outside the gel of,

(8) $$\begin{array}{c} \Pi_{ionic}=RT\Gamma_{gel}\left(c_{+,gel}+c_{-,gel}\right)-RT\Gamma_{sol}\left(c_{+,sol}+c_{-,sol}\right), \end{array}$$

where $\Gamma_{gel}$ and $\Gamma_{sol}$ are the osmotic coefficients of the gel and solution, respectively, and c are the molar concentrations of the positive (+) and negative (−) ions in the gel (gel) and in the solution (sol). Throughout this model, the gel and solution are assumed osmotically ideal and therefore, the osmotic coefficients of each are set to one. The positive and negative molar concentrations of ions inside the gel can be calculated through,

(9) $$\begin{array}{c} \begin{array}{c} c_{+,gel}+c_{-,gel}=\sqrt{\left(c_{CI}^{2}\right)+4{\left(c_{+,sol}+c_{-,sol}\right)}^{2}}, \end{array} \end{array}$$

where c${}_{CI\;}$ is the concentration of the osmotically active sodium counterions inside the gel. Therefore, the total swelling pressure in the system is derived by combining Equations (4), (5) and (7),

(10) $$\begin{array}{c} \Pi_{swelling}=-RT\left[\frac{1}{V_{m}}\left(ln\left(1-\phi_{s}\right)+\phi_{s}+\chi_{0}\phi_{s}^{2}+\chi_{1}\phi_{s}^{3}\right)-\left(c_{+,gel}+c_{-,gel}\right)+\left(c_{+,sol}+c_{-,sol}\right)\right]. \end{array}$$

#### 2.1.4. Elastic Energy

The elastic energy ceases the gel from swelling largely. The pressure of the fluid in the pores (*p*) is defined as,

(11) $$\begin{array}{c} p=\mu_{f,sol}-\Pi_{swelling} \end{array}$$

where $\mu_{f,sol}$ is the chemical potential per unit volume. The stress in the system is then calculated using the pore pressure. Utilizing continuum mechanics and the Terzaghi decomposition to break the total stress ($\sigma_{total}$) into the effective stress ($\sigma_{eff}$) on the solid matrix of the system and the fluid pressure in the pores,

(12) $$\begin{array}{c} \sigma_{total}=\sigma_{eff}-pI, \end{array}$$

where **I** is a third order identity matrix [31]. Implementing a modified neo-Hookean strain energy density function as the elastic Helmholtz free energy which was originally derived for modeling small deformation of soft porous media with a strain dependent Poisson ratio ($v=0.5\phi_{s}$, where $\phi_{s}=\;\phi_{s,0}/J$) [32,33],

(13) $$\begin{array}{c} ∆F_{elastic}=\frac{G(1+0.5\phi_{s,0}J)}{12(1-\phi_{s,0}J)}ln^{2}\left(det\left(C\right)\right)+\frac{G}{2}\left(tr\left(C\right)-3det{\left(C\right)}^{1/3}\right), \end{array}$$

where *G* is the shear modulus, $\phi_{s,0}$ is the solid volume fraction at the initial state and *J* is the Jacobian of the deformation tensor (**F**) and denotes the volume change (deformation) in reference to the initial state in a three-dimensional model,

(14) $$\begin{array}{c} J=det(F). \end{array}$$

Differentiating Equation (1) with respect to the right Cauchy Green strain (**C**),

(15) $$\begin{array}{c} \sigma_{eff}=\frac{1}{J}F\frac{\partial ∆F_{elastic}}{\partial C}F^{T}, \end{array}$$

results in the effective stress acting on the solid matrix of the gel as,

(16) $$\begin{array}{c} \sigma_{eff}=-\frac{ln(J)GI}{6J}\left[-1+\frac{3(J+\phi_{s,0})}{\left(-J+\phi_{s,0}\right)}+\frac{3ln\left(J\right)J\phi_{s,0}}{{\left(-J+\phi_{s,0}\right)}^{2}}\right]+\frac{G}{J}\left(F.F^{T}-J^{2/3}I\right). \end{array}$$

The dependency of the Poissons ratio on the deformation of the gel states as the solid volume fraction goes to zero (at fully swollen state), the system becomes fully compressible whereas in its dry state, the system is incompressible.

#### 2.1.5. Kinematics

This system of equations evolves over discretized time resulting in a transient swelling model. The positions (**x**) in any time frame (t) can be connected back to reference frame (**X**) through a mapping function ($\chi_{s}$),

(17) $$\begin{array}{c} x=\chi_{s}\left(X,t\right), \end{array}$$

which can then be used to calculate the deformation tensor (**F**),

(18) $$\begin{array}{c} F=\frac{\partial \chi_{s}}{\partial X}. \end{array}$$

As a result of an increased deformation (subbing **F** into Equation (1)), the three energies of Equation (1) are re-calculated. The effective stress (Equation (1)), is calculated as is. The mixing and ionic energies must update the solid volume fraction and counterion concentration respectively through,

(19) $$\begin{array}{c} \phi_{s}=\frac{\phi_{s,0}}{J}, \end{array}$$

and,

(20) $$\begin{array}{c} c_{CI}=c_{CI,0}\frac{\phi_{f,0}}{J-\phi_{s,0}}, \end{array}$$

where c${}_{CI,0}$ is the initial counterion concentration and $\phi_{f,0}$/$\phi_{s,0}$ are the initial fluid/solid volume fractions [26]. These values are then substituted into Equation (1) and Equation (9) respectively.

### 2.2. Finite Element Model & Model Parameters

To highlight the transient effects of including the mixing energy, finite element analysis is used. Initially, a spherical gel is investigated. Subsequently, to examine the impact on transient surface instabilities, a cylinder is considered. In order to better preserve local mass conservation compared to the standard finite element method, a Mixed Hybrid Finite Element Method (MHFEM) is implemented [10]. This method calculates the fluid flux across each element as an independent variable rather than through the numerical differentiation of the chemical potential field, hence separating their dependency on each other. This models efficacy in better replicating large deforming ionized gels has been previously reported [12].

Initially, the gel rests in a sodium chloride solution of high molar concentration conditions so that the pores are saturated without causing the swelling of the gel. Subsequently, the gel is placed in a sodium chloride solution of given molar concentration (Table 1), causing swelling. The gel is, therefore, in a stress-free rather than a dry state which negates the need of model flow into unsaturated porous media. The gel is restricted from translating or free body rotation and fluid flow can only enter the gel through the external surface. The model parameters held constant throughout each of the simulations are outlined in Table 2.

### 2.3. Solutions

Both univalent and divalent external solutions are investigated in order to analyse the effect of divalent ions on the swelling magnitude and dynamics. Pure sodium chloride (NaCl) of physiological concentrations is used for the univalent solution. For the divalent solution, calcium chloride is added to the univalent solution (NaCl + CaCl${}_{2}$). The concentrations and associated Flory Huggins parameters (χ${}_{0}$ and χ${}_{1}$) are listed in Table 1 [20].

### 2.4. Experimental Validation

To validate the implementation of the mixing energy into the numerical model against experimental data, the centrifuge method is used to measure swelling magnitude of a batch of sodium polyacrylate particles, polymerized according to EP2851048A1 [37]. The volume change of the experimental swelling is compared to that of each of the energy models (ionic, mixing & swelling). The centrifuge method takes a weight of dry polymer beads in a bag, saturates them and then removes excess fluid with a centrifugal separator [38]. The bag is then weighed again allowing for quantification of the water capacity of the gel. Here, 2 g of sodium polyacrylate was weighed and placed into a 0.9% wt. sodium chloride solution for 30 min. The weight change (known as capacity) was adjusted for density and porosity (as the initial weight measurement does not take into account the porosity of the dry polymer) in order to determine the overall volume change of the material through,

(21) $$J_{exp}=\frac{\frac{m_{gel}}{\rho_{gel}}+\frac{m_{fluid}}{\rho_{fluid}}}{\frac{m_{gel}}{\rho_{gel}}+\frac{m_{gel}}{\rho_{gel}}\left(\frac{\phi }{1-\phi }\right)}$$

where $J_{exp}$ is the experimental volume change, *m* is the mass, ρ is the density and ϕ is the porosity (the dry equivalent of the fluid volume fraction—the volume of gel not occupied by the polymer). This process is repeated for 24 batches of hydrogels (*n* = 24).

## 3. Simulation Results

In order to investigate the effect of each energy on gel swelling, numerical MHFEM simulations were conducted using the ionic pressure, the mixing pressure and the total swelling pressure (ionic + mixing pressure) for both univalent and divalent external solutions. The effect of the initial porosity (defined as 1 minus the solid volume fraction) on each swelling pressure as well as the total deformation was also analyzed.

### 3.1. Univalent Solution

**Figure 2:**
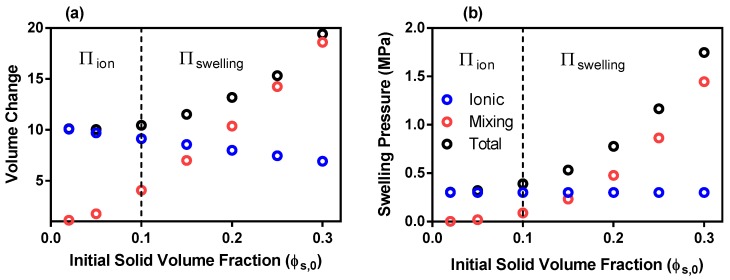
***Univalent Solution:*** (**a**) Equilibrium volume change of the ionic, mixing and total swelling pressure models as a function of initial solid volume fraction (**b**) Initial swelling pressures of ionic, mixing and total swelling energies as a function of initial solid volume fraction. [$\Pi_{ion}$ and $\Pi_{swelling}$ keys denote the swelling pressure model that should be used at each initial solid volume fraction].

The equilibrium volume change, J, (Figure 2a) resulting from the total swelling pressure (black circles) at initial solid volume fractions less than 0.10 closely match those of the ionic swelling pressure (blue circles). The ionic swelling pressure (blue circles Figure 2b) remains constant with increasing $\phi_{s,0}$, however the dependency of the elastic energy on $\phi_{s,0}$ (Equation (1)) results in a decrease of the ionic pressure model volume change (blue circles) from 15 to 10 as $\phi_{s,0}$ increases from 0.02 to 0.30. The mixing pressure increases according to the dominant term in Equation (1), $ln(1-\phi_{s,0})$, with increasing $\phi_{s,0}$ (red circles Figure 2b) which is reflected in the mixing deformation (red circles Figure 2a). This results in the mixing energy becoming the dominant swelling force at $\phi_{s,0}$ greater than 0.20. Therefore, the assumption that the ionic pressure is the sole contributor to ionized hydrogel swelling [10] can be considered correct where $\phi_{s,0}$ is less than 0.10 (which is represented by the blue region in Figure 2). However, at $\phi_{s,0}$ greater than 0.10, the mixing energy has a considerable effect on ionized gel swelling in univalent solutions and should be included in swelling models. At very large $\phi_{s,0}$ (greater than 0.5), the mixing energy can be considered the sole contributor to gel swelling with the ionic energy assumed negligible. Conventionally, this is the case in non-ionized gel swelling models [11]. Nonetheless, the $\phi_{s,0}$ of sodium poylacrylate is reported in the region of 0.17–0.31 and therefore, should include both ionic and mixing energies [10,39].

### 3.2. Divalent vs. Univalent Solutions

**Figure 3:**
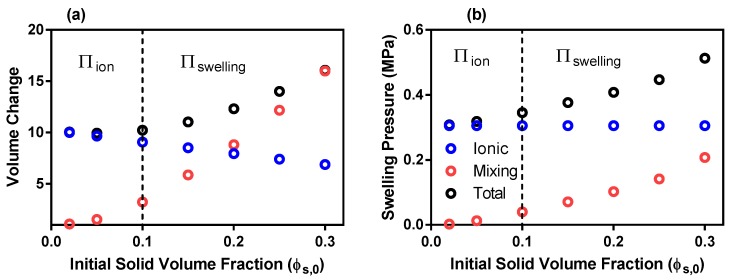
***Divalent Solution:*** (**a**) Equilibrium volume change of the ionic, mixing and total swelling pressures as a function of initial solid volume fraction (**b**) Initial swelling pressures of ionic, mixing and total swelling energies as a function of initial solid volume fraction. [$\Pi_{ion}$ and $\Pi_{swelling}$ keys denote the swelling pressure model that should be used at each initial solid volume fraction].

The inclusion of divalent ions into a univalent solution increases the concentration of charges within the solution. However, at such a small molar concentrations (see Table 1), the effect on the ionic pressure is minimal when compared to univalent solutions (blue circles Figure 2b and Figure 3b). The results of the mixing swelling pressure model are represented by the red circles in Figure 3. For an $\phi_{s,0}$ of 0.20, the initial mixing pressure reduces from 0.45 MPa for univalent solutions (Figure 2b) to only 0.1 MPa for divalent solutions. The effect of the squared and cubic terms in Equation (1) are diminished due to the larger second Flory–Huggins parameter (Table 1). However, the rate of decay of the mixing pressure is also abated and as a result the mixing energy has a greater influence on gel swelling at later time points. As a result, the deformations experienced by the divalent solution model was only slightly less than that of the univalent solution (black circles Figure 2a and Figure 3a). So although the mixing pressures influence on the initial total swelling pressure of the divalent solution is much less than that of the univalent solution, it is still necessary to include them in the swelling model.

**Figure 4:**
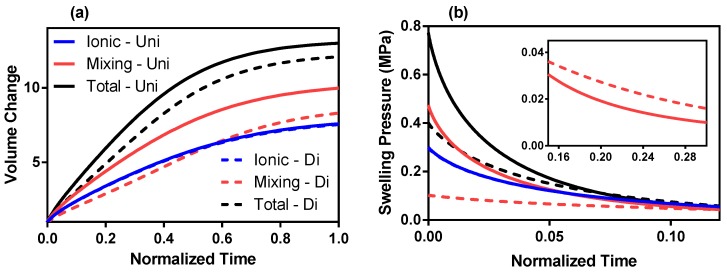
(**a**) Volume change as a function of normalized time (fraction of time to reach equilibrium) of ionic, mixing and total swelling pressures for univalent and divalent solutions. (**b**) Swelling pressures of ionic, mixing and total swelling energies of univalent and divalent solutions as a function of normalized time. $\phi_{s,0}$ = 20%. Inlay: Mixing energy swelling pressure (MPa) between 0.15 and 0.3 of normalized time.

When comparing the univalent and divalent solutions side by side, the deformation of the divalent model lags behind that of the univalent model and reaches equilibrium at a slightly lower value (total swelling model: divalent—18, univalent—19 for $\phi_{s,0}$ = 0.20) (Figure 4a). This lag is not present in the ionic pressure model as the Flory–Huggins parameters are not included in the model. However, it is more prominent in the mixing pressure model as the total swelling pressure model averages out the lag between the mixing and ionic pressure models. Therefore, the mixing pressure model is more susceptible to deformation reduction than the ionic pressure and total swelling pressure models.

The difference in initial swelling pressures between univalent and divalent solutions is much larger than the difference between equilibrium deformations for both the mixing and total swelling pressure models. Again, the ionic pressures are identical for both solutions (blue dashed and blue full lines Figure 4b). The initial mixing pressure of the univalent solution is much greater than that of the divalent solution (0.45 MPa compared to 0.1 MPa). However, the univalent pressure drops below the divalent pressure within the first 10% of the time to swelling equilibrium. After that, the divalent mixing pressure is greater than the univalent until swelling equilibrium is reached as seen in the subplot of Figure 4b. This results in the equilibrium deformation difference between univalent and divalent solutions not being as drastic as the initial swelling pressure difference. This phenomenon translates to the total swelling pressure through Equation (1).

**Figure 5:**
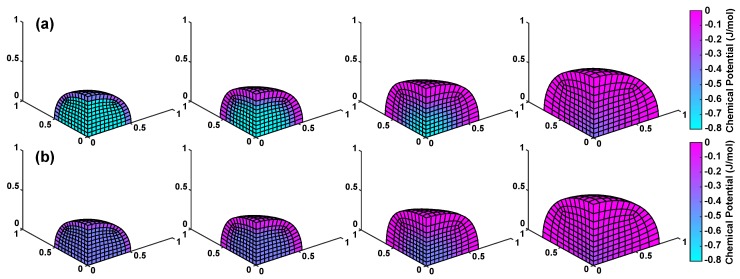
Chemical potential (μ) difference between inside the gel and the external solution at increasing time points (15%, 43%, 67% & 92% of equilibrium time) for (**a**) univalent and (**b**) divalent solutions for the total swelling pressure model. $\phi_{s,0}$ = 20%. Note: the chemical potential difference is equal in magnitude to the swelling pressure difference.

Analyzing the spatial distribution of the chemical potential difference between inside and outside the gel highlights the difference in initial swelling pressure between univalent and divalent solutions (Figure 5a, Figure 5b respectively). The core of the univalent solution model remains light blue until the last time point. However, comparing the 55% to equilibrium time graphs, the gradient across the univalent is much steeper. The divalent model has a much smoother profile resulting in slower swelling.

As mentioned, the independence of the three energies of the FR theory are not exact. Particularly for these simulations, the influence of the ionic energy on the mixing pressure is of importance. The presence of ionized polymer sub-units making up the polymer backbone coupled with a certain degree of neutralization of these charges results in an influence on how the solvent mixes with the polymer [15]. This generates a complicated relationship dependent on counterion and external molar concentration as well as swelling dynamics and temperature. However, the influence of ionic constituents on the Flory–Huggins parameters of sodium polyacrylate are minimal for univalent solutions. Moreover, the influence of this relationship in divalent solutions is overshadowed by the cross-linking/charged group interaction effect of divalent ions, evident in the lower mixing pressures than univalent solutions [20]. Although, a relationship between these pressures exists, it has been shown that the interactions between the energies of Flory–Rehner does not play a significant role on swelling magnitude, especially at physiological salt concentrations, and therefore, this study assumes they are independent [19,40].

### 3.3. Transient Surface Instabilities

Transient surface instabilities are defined as the geometrical wrinkling of the surface during swelling. To investigate the effect of the mixing energy inclusion on this phenomenon, MHFEM simulations are conducted on a cylindrical geometry (diameter = 2 mm, height = 1 mm) with and without the mixing energy in a 0.3% wt. NaCl solution (to emphasize wrinkling).

**Figure 6:**
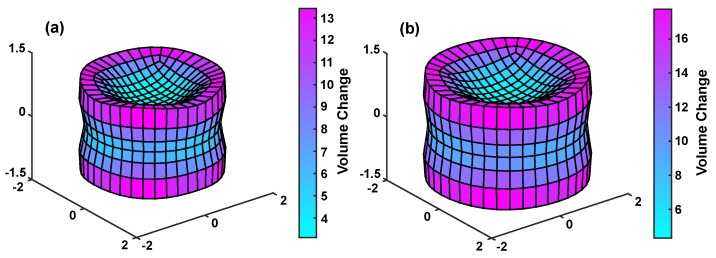
Transient surface instabilities of (**a**) ionic pressure simulation and (**b**) total swelling pressure simulation after 35 s. (Full videos available at http://dx.doi.org/10.17632/95svb4kr2j.1).

Figure 6 displays the morphology of the cylinder at 35 s for just the ionic pressure (a) and the total swelling pressure (b). The increased pressure of the total swelling model causes a larger deformation than the ionic model at this time point (4.83 vs. 7.36). Following this, although the geometries are similar, the instability magnitude in the total swelling model is greater than the ionic model. The differences between the largest deforming and smallest deforming element on the surface (a measure of surface instabilities) of each is 10.30 and 13.45, respectively. To validate the instabilities, qualitative comparisons are made to a cylindrical pHEMA/polyacrylamide gel swelling experiment in a de-ionized water solution (videos available at http://dx.doi.org/10.17632/95svb4kr2j.1). The concave instability morphology on the side face of the cylinder (first 10 s of the video) in the experiment is repeated for both the total swelling and ionic model simulations. Subsequently, the gel deforms to an irregular geometry which cannot be replicated in the current MHFEM because of uncertainty of the mechanical parameters. Finally, it returns to a cylindrical geometry again, much like both simulations. It is difficult to hypothesize which simulation is more representative without a more quantitative methodology as well as the prescription of perfect material properties. However, these videos together with Figure 6 highlight the effect of including the mixing energy on the transience of the process.

## 4. Comparison to Theory

For numerical verification of the model presented here, the results obtained are compared to the theoretical predictions of the ionic, mixing and total pressures for varying deformation values. The mixing pressure is calculated with increasing deformation by updating the solid volume fraction ($\phi_{s}$) in Equation (1).

**Figure 7:**
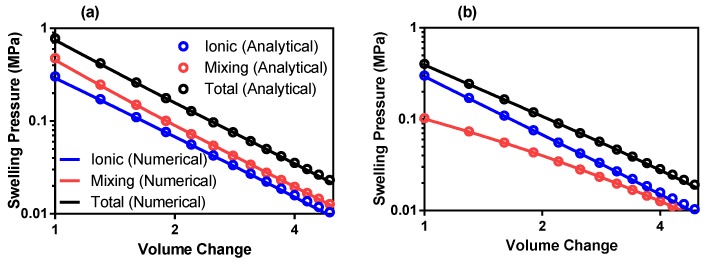
Model comparison to theoretical predictions of (**a**) univalent and (**b**) divalent solutions at $\phi_{s,0}$ = 20%. Note: both axes are represented by a log scale for simplicity of visualization of a linear function.

The updated solid volume fraction is then subbed into the Flory–Huggins equation (Equation (1)). To update the ionic pressure with increasing deformation, the updated counterion concentration (c${}_{CI}$) is calculated through Equation (1) and is subbed into Equation (9). Theoretical predictions for both ionic and mixing energies are calculated at intervals of 0.03 between deformations (J) of 1 and 10 at an initial solid volume fraction of 0.20. The total swelling pressure is taken as the addition of ionic and mixing pressures (as per Equation (1)). Only the outer layer of elements in constant contact with the external solution of the model are considered for theoretical validation. Otherwise, the theory must also consider the permeability of the model. This procedure is repeated for both univalent and divalent solutions with the results presented in Figure 7.

From Figure 7, the numerical model fits well with the theoretical description of the process for both univalent and divalent solutions. Deviations from the ideal theoretical values can be attributed to the irregularity of the element sizes on the outer layer of the geometry. This is due to the difficulty in uniformly meshing a sphere (or an eighth of a sphere). The root-mean-square error (RMSE) is calculated between the theoretical and model results. The results of the errors are displayed in Table 3 showing good agreement.

## 5. Experimental Validation

**Figure 8:**
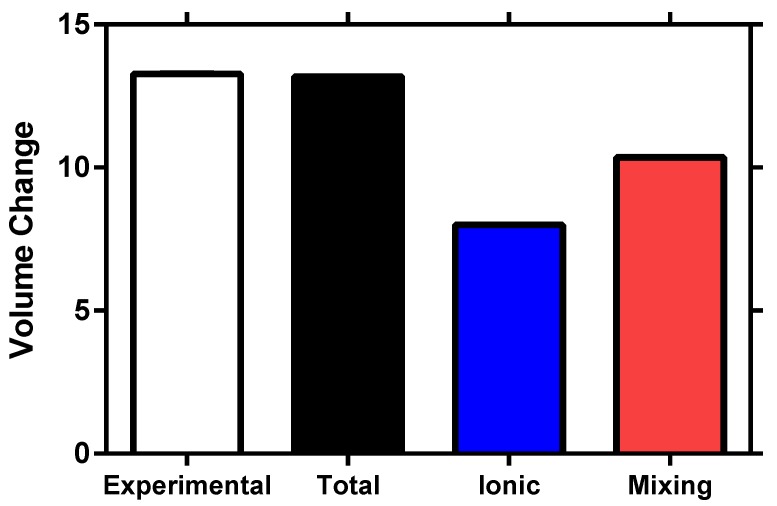
Experimental volume change results (*n* = 24) compared to the total swelling pressure, ionic pressure and mixing pressure models in a univalent solution. The total swelling model shows greatest fit with experimental data, hence validating the addition of the mixing energy to the ionized hydrogel numerical swelling model.

For exhaustive validation of the proposed ionized superabsorbent polymer swelling model incorporating the mixing energy of the Flory–Rehner theory, comparison of the experimental and numerical volume change magnitudes is conducted. Figure 8 displays the results of the experimental swelling tests as well as the volume changes of the ionic pressure, mixing pressure and total swelling pressure models. As the total swelling pressure model is within 0.5% of the experimental results, with the ionic and mixing models at 39.6% and 21.8% difference respectively, the inclusion of the mixing energy into the swelling model is essential for accurate volume change magnitude simulations. The individual energy models (ionic and mixing) do not reach the magnitude of the experimental swelling tests and therefore, should not be implemented individually into a swelling model replicating ionized superabsorbent polymer deformation.

## 6. Conclusions

Previous studies have highlighted the insignificance of the mixing proportion of the total free energy within the Flory–Rehner theory when modeling large deformation of ionized hydrogels. Here, full three-dimensional transient numerical simulations of sodium polyacrylate swelling with the inclusion of the mixing energy described by the Flory–Huggins equation are presented. From the model results, the mixing energy significantly affects the swelling magnitude of ionized hydrogels at initial solid volume fractions greater than 0.10 and, therefore, it should be included in the model. At initial solid volume fractions lower than 0.10, the mixing energy can be assumed negligible for both univalent and divalent solutions as its effect on swelling magnitude is minimal. This assumption is in good agreement with deswelling experiments conducted on sodium polyacrylate [20]. However, the initial solid volume fraction of sodium polyacrylate lies in the range of 0.15–0.30. For divalent solutions, the deformation lags slightly behind that of univalent solutions. Nonetheless, the swelling magnitude is not significantly different between the two solutions. This contradicts the fact that the initial swelling pressures are much greater for univalent solutions over divalent solutions. The results presented here signify the importance of the average swelling pressure over the course of the swelling rather than the initial peak pressure. Furthermore, focusing on surface instabilities, the inclusion of the mixing energy produced a significant difference in the timing of surface morphological changes. Finally, to verify the results presented here, comparison with an analytical solution as well as experiments was performed, showing the inclusion of the mixing energy is essential in replicating real-world volume change magnitudes. Despite the findings of this study, some limitations should be noted. Firstly, the gel and solution are assumed to be osmotically ideal. Yet, the osmotic coefficient of sodium polyacrylate is extremely dependent on the concentration of polymer, degree of neutralization and cross-link density [41] while the osmotic coefficient of a sodium chloride solution is considered to be 0.93 [42]. Secondly, the external concentrations used are severely limited to those used in the experiments to calculate the Flory–Huggins parameters. Extending these simulations to non-physiological external salt concentrations would require repeat of deswelling experiments to determine the associated Flory–Huggins parameters. However, previous studies have shown minimal difference in these parameters across different concentrations of univalent solutions [20]. Therefore, reducing the external concentration of a univalent solution will cause an overall increase in swelling magnitude predominately caused by an increase in ionic osmotic pressure, with the pressure associated with mixing remaining unchanged. The opposite trend holds true for an increase in external concentration, with the ionic osmotic pressure reduction leading to a reduction in swelling magnitude. Divalent solution changes influence the Flory–Huggins more significantly, especially $\chi_{1}$, leading to a more substantial effect of the mixing energy on swelling magnitude. Overall, this study has proven that the mixing energy is essential when modeling the transient swelling of sodium polyacrylate in both univalent and divalent solutions.

## Figures and Tables

**Table 1:** List of parameters dependent on the external solution and their associated values.

Parameter	NaCl	NaCl + CaCl${}_{2}$
Flory Huggins Parameter 1 ($\chi_{0}$)	0.45	0.453
Flory Huggins Parameter 2 ($\chi_{1}$)	0.21	0.53
Molar Concentration	0.154 M	0.154 M + 8 × 10${}^{-4}$ M

**Table 2:** List of model parameters, which remain constant throughout each simulation and their associated values. Permeability parameter (β) referes to a strain dependent permeability constant [34,35,36]. The value of this constant does not affect the magnitude of the swelling but rather the time taken to reach swelling equilibrium.

Parameter	Value
Shear Modulus	18 kPa
Degree of Neutralisation	0.85
Initial Counterion Concentration	0.3 M
Permeability Parameter (β)	2
Temperature	298 K
Universal Gas Constant	8.3145 J/K.mol
Solvent Molar Volume	18 mol/m${}^{3}$
Initial Hydraulic Permeability	10${}^{-3}$ mm${}^{4}$/Ns

**Table 3:** Root Mean Square Error (RMSE) between theoretical predictions and simulation results.

Model	RMSE (MPa)
Univalent—Total Swelling Pressure	0.085
Univalent—Ionic Pressure	0.037
Univalent—Mixing Pressure	0.047
Divalent—Total Swelling Pressure	0.069
Divalent—Ionic Pressure	0.054
Divalent—Mixing Pressure	0.016

## Abbreviations

The following abbreviations are used in this manuscript:

$CaCl_{2}$	Calcium Chloride
MHFEM	Mixed Hybrid Finite Element Method
NaCl	Sodium Chloride
pHEMA	Polyhydroxyethylmethacrylate
